# Curated Collection for Educators: Five Key Papers About Impostor Phenomenon

**DOI:** 10.7759/cureus.88741

**Published:** 2025-07-25

**Authors:** Joshua Ginsburg, Marshall Howell, Erin Lincoln, Vivek Medepalli, Joshua Gentges, Daniel M Courtney, Brian Milman

**Affiliations:** 1 Emergency Medicine, The University of Texas (UT) Southwestern Medical Center, Dallas, USA; 2 Emergency Medicine, The University of Oklahoma Health Sciences Center, Tulsa, USA

**Keywords:** coaching, faculty development, impostor phenomenon, medical education, transition points

## Abstract

Impostor phenomenon (IP) is characterized by persistent doubt in one’s skills and accomplishments and a fear of being exposed as a fraud. It has been recognized as a significant challenge across various fields, including emergency medicine (EM), as IP has been linked to burnout, career stagnation, and adverse impacts on mental health. It is therefore vital that faculty development efforts target recognition of IP and mitigation of its negative effects on learners. However, given the vastly growing body of literature on IP, it may be difficult for educators to identify the most relevant resources to guide these efforts. Using a modified Delphi process, we curated a collection of literature to guide EM educators seeking to better understand and address IP. These included a qualitative study on physicians’ self-assessment, a scoping review of IP literature, and studies examining structural contributors to and protective interventions against IP.

## Introduction and background

Impostor phenomenon (IP), also known as impostor syndrome, is a psychological experience in which individuals doubt their own skills or accomplishments and fear being exposed as frauds. First described among high-achieving women by Clance and Imes in 1978, IP is now known to be prevalent among highly successful individuals in other fields, including emergency medicine (EM), in which over 80% of both men and women have been reported to have moderate to significant IP feelings [1,2]. Several factors may lead to IP in EM. Emergency physicians (EPs) are responsible for the initial phase of care for patients with any complaint, which requires proficiency in a comprehensive range of clinical and procedural skills. Additionally, EPs must always be prepared for the resuscitation of undifferentiated, critically ill patients. These conditions require a substantial breadth of knowledge, frequent interactions with consultants and specialists, efficient management of time and physical resources, and the ability to manage stress and uncertainty. These demands may exacerbate feelings of inadequacy commonly associated with IP. 

IP is increasingly recognized as more than just an inconvenient or fleeting thought process; it has been associated with career stagnation, burnout, anxiety, and suicidal ideation [3-5]. Educators must recognize the signs of IP and develop interventions to mitigate its negative impacts on learners. To this end, literature in medicine and medical education has explored specialty-specific and environmental factors contributing to IP, qualitative aspects of IP, and methods to support learners who experience IP. Given this growing body of scholarship, it may be difficult for educators to quickly identify the most relevant and practical resources to guide their understanding of IP and how to combat it. 

This curated collection highlights five key papers about IP and is intended to serve as a foundational reading list for EM educators interested in learning more about the topic. These papers were selected for their thoughtful and thorough explorations of the prevalence of IP, the contributors to and lived experience of IP, and evidence-based strategies for combating IP.

## Review

Methods

In September 2024, The University of Texas (UT) Southwestern Medical Center's Department of Emergency Medicine organized a Scholarship Incubator program. This incubator organized faculty members with similar scholarship interests into three communities of practice. This author group focused on medical education scholarship. 

The group was intentionally composed to represent a diverse range of academic experiences and ensure that both early-career and senior educators could identify papers meaningful to different stages of academic development. The group included a current medical education fellow, two junior faculty members focused on medical education, a previous Associate Program Director, the Medical Education Fellowship Director, and the Executive Vice Chair of the department. To add geographic and institutional diversity, the Research Director from another academic institution was included in the group. All members were trained at different EM residency programs. 

**Figure 1:**
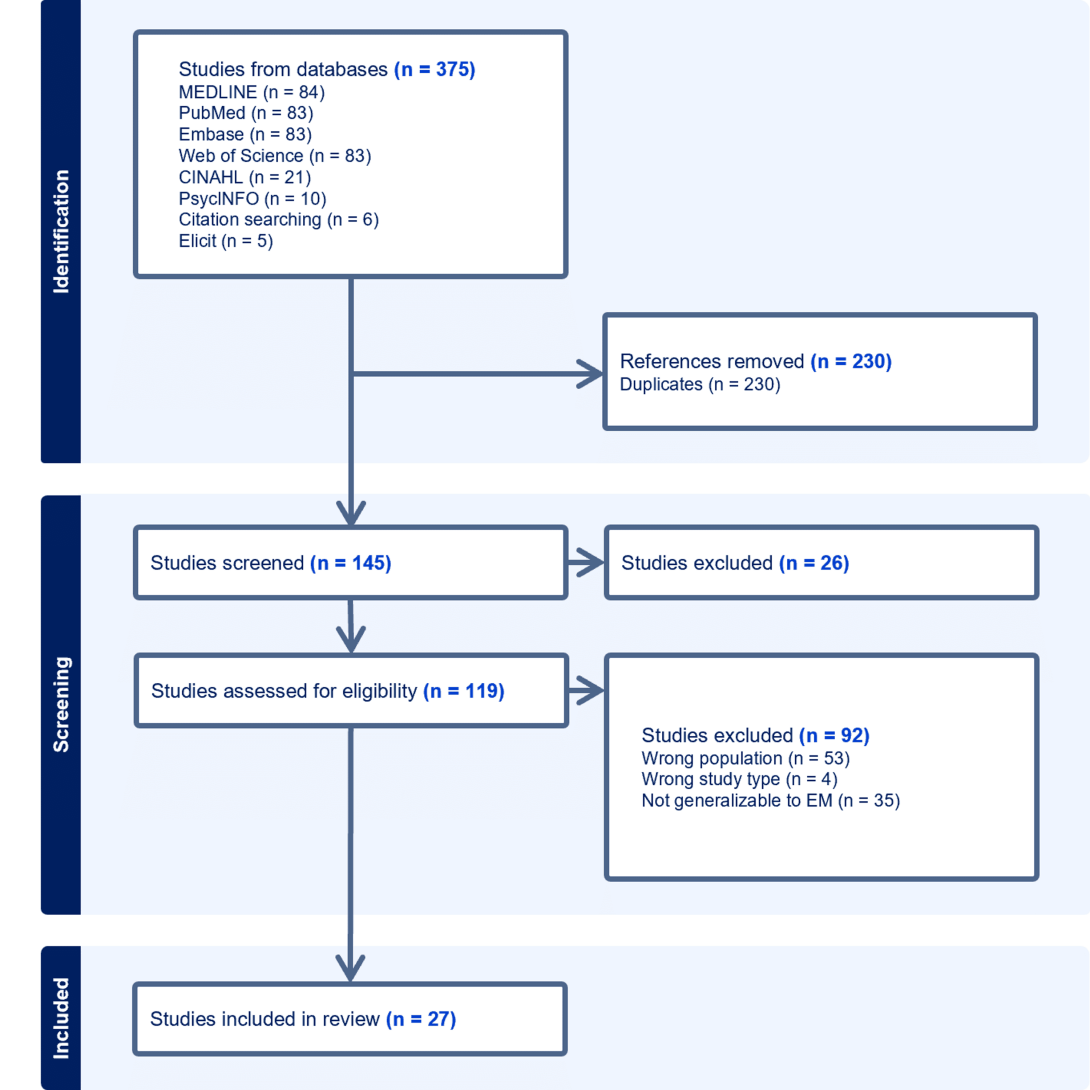
PRISMA flow diagram of study selection PRISMA: Preferred Reporting Items for Systematic reviews and Meta-Analyses; EM: Emergency medicine

To identify relevant articles, BM performed a search on MEDLINE, PubMed, Embase, Web of Science, CINAHL, and PsycINFO. The final search was performed in January of 2025. Only English-language articles were included. All dates were included. The full search query is included in the Appendices. Additionally, JG, MH, EL, and VM conducted independent searches in PubMed and Google Scholar. Additional articles were included by reviewing the references of the included articles. Articles were compiled using Zotero (Center for History and New Media, George Mason University, Fairfax, United States), a reference management software that allows group members to collect, organize, and annotate articles. To ensure that no key articles were missed, the search was augmented using Elicit (Ought, San Francisco, United States), an artificial intelligence (AI) tool that identifies “seed articles.” The Preferred Reporting Items for Systematic reviews and Meta-Analyses (PRISMA) diagram is presented in Figure 1. 

To reach consensus on the most impactful papers for medical educators, we employed a three-round voting process based on Delphi methodology. This process follows the precedent of multiple papers published through the Academic Life in Emergency Medicine Faculty Incubator [6]. Following the literature review, the group collaboratively developed the rating system to be used in Round 1: a seven-point Likert scale anchored at 1 by “not at all relevant to emergency medicine faculty learning about impostor phenomenon,” at 4 by “moderately relevant to emergency medicine faculty learning about impostor phenomenon,” and at 7 by “essential to emergency medicine faculty learning about impostor phenomenon.” Each group member rated all articles using the seven-point Likert scale. In Round 2, group members reviewed a frequency histogram showing how members rated each article in Round 1. They then categorized each article as “should be included in top papers” or “should not be included in top papers.” There was no limit to the number of articles that could be included in either category in Round 2. In Round 3, group members were shown the percentage of members who selected each paper for inclusion. The results of each round were anonymized to encourage free expression and minimize bandwagon effects. All articles with at least 50% endorsement in Round 2 remained in consideration. Each member was invited to advocate for the five papers they felt should be included in the final manuscript. Equal consideration was given to each member's opinion, and dissenting opinions were encouraged. Discussion started with junior faculty and progressed to senior faculty to limit authority bias. Chosen papers were required to receive a minimum of five votes for inclusion. 

This process does not follow the traditional Delphi methodology, since we included both junior and senior faculty. However, this was done intentionally to ensure that the final selection of articles would be relevant across varying levels of experience in medical education. 

Results

Group discussions, literature search, and Elicit search yielded 27 articles relevant to IP in EM. A total of 14 papers received “should be included in top papers” votes in Round 2, with nine receiving votes from at least 50% of the group. In Round 3, three papers received endorsement by 100% of the group and were included in the final manuscript. Five of the remaining six papers in consideration received votes for inclusion in Round 3, with the two highest-scoring papers receiving endorsements from 85.7% of the group. These papers were included as the final selections for the manuscript. Each paper is summarized and discussed in the discussion section below. Ratings of the 27 papers reviewed are included in Table 1. 

**Table 1:** Complete list of IP literature compiled by the authorship team and results from each round of the modified Delphi process IP: Impostor phenomenon; SD: Standard deviation

Citation	Round 1	Round 2	Round 3	Rank
	Initial mean scores (SD), max score 7	% of raters who endorsed this paper	% of raters who endorsed this paper	
					LaDonna et al. [3]	6.1 (0.7)	100.0%	100.0%	1
Gottlieb et al. [5]	6.0 (1.2)	100.0%	100.0%	2
Chodoff et al. [7]	5.3 (1.3)	100.0%	100.0%	3
Shanafelt et al. [8]	5.3 (1.6)	71.4%	85.7%	4
Rehsi and McCarthy [9]	5.7 (1.7)	85.7%	85.7%	5
Siddiqui et al. [10]	5.7 (1.0)	85.7%	14.3%	
Morgenstern and Beck Dallaghan [11]	5.1 (2.3)	57.1%	0.0%	
Batur et al. [2]	5.0 (1.7)	14.3%	0.0%	
Rivera et al. [12]	4.9 (1.6)	71.4%	0.0%	
Thomas and Bigatti [13]	4.9 (1.1)	0.0%	0.0%	
Franchi and Russell-Sewell [14]	4.7 (1.7)	42.9%	14.3%	
Clance and Imes [1]	4.6 (1.1)	71.4%	14.3%	
Freeman and Peisah [15]	4.6 (1.0)	28.6%	0.0%	
Ogunyemi et al. [16]	4.4 (0.8)	28.6%	0.0%	
Bravata et al. [4]	4.4 (1.5)	0.0%	0.0%	
Clance and OToole [17]	4.4 (1.5)	14.3%	0.0%	
Gottlieb [18]	4.1 (1.6)	0.0%	0.0%	
Neufeld et al. [19]	3.9 (1.2)	0.0%	0.0%	
Villwock et al. [20]	3.7 (1.3)	0.0%	0.0%	
Legassie et al. [21]	3.6 (1.4)	0.0%	0.0%	
Rosenthal et al. [22]	3.6 (1.0)	0.0%	0.0%	
Jefferson et al. [23]	3.1 (1.2)	0.0%	0.0%	
Chakraverty et al. [24]	3.0 (1.0)	0.0%	0.0%	
Joseph et al. [25]	3.0 (1.5)	0.0%	0.0%	
Leach et al. [26]	3.0 (1.4)	0.0%	0.0%	
Bhama et al. [27]	2.7 (1.0)	0.0%	0.0%	
Stephens [28]	1.4 (0.5)	0.0%	0.0%	

Discussion

Paper 1

LaDonna KA, Ginsburg S, Watling C: "Rising to the level of your incompetence": what physicians’ self-assessment of their performance reveals about the imposter syndrome in medicine. Acad Med. 2018, 93:763-8 [3]

Summary: This qualitative study explored the presence of self-doubt and IP among physicians of varying clinical specialties. Using semi-structured telephone interviews, the authors examined feelings of inadequacy and self-doubt among 28 physicians at varied stages of training and years of practice. One prominent theme was the wide spectrum of self-doubt: some participants described it as rare, others experienced it persistently, and most reported intermittent episodes. Additionally, participants remarked that the self-doubt they experienced was recurrent and often associated with periods of high stress. They also noted a disconnect between the internal perception of performance and the feedback received. While the feedback was often positive and validating, many individuals had difficulty accepting the praise and felt that it was inaccurate. The authors argued that medical culture itself perpetuated these feelings due to the belief that “needing help does not fit with the self-image of young doctors.” 

Relevance to junior faculty members: This article provides discussion points that are crucial to junior faculty, who often experience many of these feelings as they transition from the role of trainee to attending physician. It acknowledges the common feelings of self-doubt and IP across various stages of training and clinical specialty, allowing junior faculty to understand that these feelings are natural as they start their careers. The article suggests that these feelings are not isolated to individuals but rather represent a pervasive phenomenon within medicine, experienced by many across specialties and stages of training. Further, it highlights that positive feedback alone is inadequate to combat feelings of IP. Junior faculty should seek mentorship opportunities and develop methods for reflection and coping. 

Considerations for faculty developers: This article offers valuable, actionable methods for faculty developers to assist in mitigating IP in junior faculty members and trainees. It acknowledges the common experience of self-doubt, inspiring more senior faculty to develop quality mentorship opportunities. Specifically, mentorship acknowledging feelings of vulnerability during periods of transition, such as from resident to junior faculty or even faculty transitioning to more senior roles, may be particularly effective. These individuals often revert to feeling like frauds, even in the face of positive feedback, and intentionality on the part of senior faculty can help junior faculty feel more supported during periods of uncertainty.

Paper 2

Gottlieb M, Chung A, Battaglioli N, Sebok-Syer SS, Kalantari A: Impostor syndrome among physicians and physicians in training: a scoping review. Med Educ. 2020, 54:116-24 [5] 

Summary: This scoping review summarized the existing literature on IP among medical students, residents, and attending physicians. Gottlieb et al. identified 18 studies that met their criteria, most of which were conducted in the United States and used the Clance Impostor Phenomenon Scale (CIPS) to determine the prevalence of IP. Prevalence rates ranged from 22% to 60%, with several studies reporting higher rates in women. Contributors to IP included perfectionism, low self-esteem, and the hierarchical culture of medicine. Protective factors against IP included social support, validation, affirmation, institutional support, and reflective practice. Most studies found that IP was associated with anxiety, depression, and burnout. The paper identified gaps in the literature, including few studies on attending physicians and the influence of practice experience on IP, as well as limited literature on the influence of sex, gender, race, and ethnicity on IP. The authors noted that there is a need to better understand IP within the medical field. They called for future research on effective preventative strategies, prospective studies of interventions, and improved assessment tools. 

Relevance to junior faculty members: This review is relevant to junior faculty members both as educators of physicians-in-training and as potential victims of IP themselves. Junior faculty who are involved in resident or medical student education should note factors that may contribute to IP in their learners, the effects it may have, and the opportunities for future research on early identification and intervention. Additionally, junior faculty members may find themselves vulnerable to IP as they take on new roles or transition from training to independent practice. This review can help normalize these feelings and should serve as encouragement to seek support and adopt practices, such as reflective practice, that may mitigate the negative effects of IP. 

Considerations for faculty developers: This review provides faculty developers with an overview of IP among physicians and physicians-in-training that can guide the development of support systems and design of future research. Specifically, the paper highlights the need to normalize IP in our profession, organize faculty development workshops that address IP, and train faculty to recognize and combat IP in learners. For both faculty and trainees, interventions can focus on identified protective factors, including positive affirmation, validation of success, and reflective practice. Additionally, faculty developers can encourage faculty to pursue research that addresses the identified gaps in the literature, including validation of tools in the medical setting and prospective studies of interventions. Moreover, as an institution, it is incumbent upon us to create psychologically safe environments that do not demand perfection from faculty or learners.

Paper 3

Chodoff A, Conyers L, Wright S, Levine R: "I never should have been a doctor": a qualitative study of imposter phenomenon among internal medicine residents. BMC Med Educ. 2023, 23:57. 10.1186/s12909-022-03982-8 [7] 

Summary: This study qualitatively explored the experience of IP during internal medicine residency. The authors conducted one-on-one semi-structured interviews with 28 residents from a single residency program. Coding of interview transcripts revealed that IP was prevalent among these trainees, and participants had a mean CIPS score of 63, consistent with frequent feelings of impostorism. Participants reported persistent feelings of inadequacy that adversely affected their learning. IP was both triggered and exacerbated by comparisons to the performance of others. Specific learning contexts intensified IP as well, including frequent role transitions with increasing responsibilities and rigid hierarchical structures. Participants described establishing unrealistic self-expectations, which resulted in disproportionate self-blame for adverse outcomes and difficulty internalizing positive feedback. They also noted that energy diverted toward projecting an appearance of competence and confidence often detracted from learning opportunities. The authors concluded by emphasizing the need for institutional awareness of IP among trainees and structural changes to the clinical learning environment to better support residents struggling with these feelings. 

Relevance to junior faculty members: While the subjects of this study were internal medicine residents, the conclusions and valuable insights into the experiences of IP are also relevant to both EM trainees and junior faculty. The study highlights IP as a widespread experience that can be a response to environmental factors, rather than personal failings. The transition from learning to a teaching role bears many similarities to the rapid changes in responsibility throughout residency and can breed self-doubt. Junior faculty adjusting to their new roles may also suffer from IP through frequent comparisons to others. Junior faculty who recognize these similarities to the resident experience are uniquely positioned to support trainees experiencing IP. One of the authors’ suggestions for addressing IP that can be implemented by junior faculty is to establish psychological safety in the clinical space. The authors suggest several ways to work toward this goal, including normalizing uncertainty, modeling vulnerability, and candidly discussing the challenges of residency and medical practice. These strategies can help both learners and junior faculty navigate IP in their changing roles. 

Considerations for faculty developers: This study highlights the social and environmental factors that contribute to IP in medical training and offers practical strategies for faculty developers to help affected learners. Impostorism is prevalent in medicine due to the performance-focused standards that demand expertise and certainty, even from junior learners. These unrealistic expectations can lead to feelings of inadequacy, excessive self-blame, and difficulty internalizing feedback. The authors suggest two primary approaches for faculty developers to address IP. First, cultivating a culture of psychological safety within the learning space and broader department can counteract the self-critical thinking of IP and promote a growth mindset. Second, faculty training on delivering frequent, thoughtful feedback and coaching can help correct distorted self-expectations among learners. The authors discuss the 'debriefing with good judgment' approach, which encourages faculty to first explore residents’ perspectives on their performance to separate their actions from preconceived negative emotions [29]. By equipping faculty with these coaching skills, institutions can help trainees recognize and reframe self-deprecating thought patterns, reducing the impact of IP. 

Paper 4

Shanafelt TD, Dyrbye LN, Sinsky C, et al.: Imposter phenomenon in US physicians relative to the US working population. Mayo Clin Proc. 2022, 97:1981-93 [8] 

Summary: In this cross-sectional study, Shanafelt et al. explored the correlation between IP and burnout, suicidal ideation, and professional fulfillment in over 3,000 physicians using the CIPS. They also compared physician disappointment with professional accomplishments to a representative sample of the general population. The authors found that IP was associated with burnout, suicidal ideation, and decreased professional fulfillment among physicians. Physicians also reported greater disappointment in their accomplishments compared to both the public and a subset of the population with doctoral or equivalent degrees. Women in medicine had significantly higher IP scores, as did younger physicians and academic physicians. The authors hypothesized that a culture of perfectionism and isolation among physicians contributed to IP and suggested interventions such as normalizing help-seeking behavior and implementing Colleagues Meeting to Promote and Sustain Satisfaction (COMPASS) groups, which provide structured peer support to reduce isolation and foster connection, thereby potentially mitigating IP. 

Relevance to junior faculty members: As IP scores are higher in younger physicians, junior faculty must be mindful of the risks IP poses both to themselves and their learners. Contributing to a just culture, avoiding a mindset of perfectionism, and modeling self-care may all reduce inappropriate feelings of inadequacy. Junior faculty should engage with IP interventions, including small group discussions with senior faculty, junior faculty, and learners. As there is evidence that IP develops early in medical school and persists after training ends, there is an opportunity to develop early interventions during the preclinical years. It is plausible that these interventions could reduce IP, which may reduce burnout and improve professional fulfillment, although current literature on the outcomes of these strategies is not well developed [10]. 

Considerations for faculty developers: EPs had the third-highest IP scores among medical specialties in the study, and scores were also higher among younger faculty. As scores rise, feelings of depersonalization increase while professional fulfillment decreases. Junior faculty in EM are therefore at higher risk of burnout and suicidal ideation, and career development could be adversely impacted by IP in this cohort. As IP is an internal process, senior faculty may not be aware of the severity or impact of IP on their junior faculty. Proactive work to assess for and counter IP could improve IP in this group, with implications for faculty retention, productivity, job satisfaction, and teaching efficacy. 

Paper 5

Rehsi AS, McCarthy KE: Twelve tips for recognizing and supporting medical learners experiencing impostorism. Med Teach. 2024, 46:489-94 [9] 

Summary: This paper provided actionable strategies for educators, educational programs, and institutions to identify and support medical learners experiencing IP. The recommendations were based on both a review of the literature and the authors’ professional experiences. While not all interventions were validated, they were research-informed and grounded in best practices. For example, prior research indicated that IP emerges most intensely at the beginning of new roles, responsibilities, and projects [30]. Thus, the authors recommended being aware of the complex and multifaceted sources of IP in students during transition points (e.g., year within residency or expectations of the clinical environment). The 12 tips provided were applicable at both the individual educator level as well as the educational program level. The authors recommended building inclusive environments, recognizing and teaching at the individual learner’s level, providing targeted and example-based feedback, acknowledging the interplay between IP and other mental health conditions, and providing general support and mentoring. 

While this article is heavily informed by expert opinion, it remains one of the few from our selection that provides specific, actionable items that allow educators to identify, address, and prevent IP. 

Relevance to junior faculty members: While many articles discuss the principal features of IP, Rehsi and McCarthy combine experience with evidence to make recommendations that educators can immediately incorporate into their practice. Many of the recommended interventions can and should be applied at a system level, but they also require individual educator buy-in. For example, creating a culture that promotes an individualized growth mindset can be an organizational goal, but it must be reinforced on an individual basis in daily interactions with learners. Additionally, it is important to recognize and address destructive habits often seen in learners with IP. Identifying perfectionism and anxiety among learners may only be possible in one-on-one or small group learning environments, and faculty should be aware of external manifestations of these conditions. 

Considerations for faculty developers: The tips in this article can be used to inform faculty development education. For example, one recommendation is to “give constructive feedback that is focused and driven by examples.” Many faculty members may not know how to provide this type of feedback. Faculty development sessions focused on delivering clear, example-driven feedback would help meet this objective. The authors also suggest setting consistent external expectations for learners that correlate with their ability level. While experienced faculty may have this skill, newer faculty may struggle to determine the ability level of a particular learner. Finally, since IP can affect learners and faculty alike, these tips can be used both for targeted faculty development and finding ways to support junior faculty members. 

Limitations

In our initial literature search, the authors identified papers deemed pertinent to the topic of IP to generate a list of articles for review. However, this was not an exhaustive list of all articles on IP, and our exclusion criteria may have omitted relevant literature in other languages or outside our date range. Our findings may have been influenced by publication bias, potentially overestimating the prevalence of IP. Additionally, since our authorship consisted of faculty with varying experience in IP, this may have introduced bias through article selection. While the traditional Delphi method utilizes only experts in a field, the use of junior faculty may have introduced bias in the process, as junior faculty in the final round of article selection may have felt influenced by more senior faculty. We attempted to mitigate this bias by anonymizing voting results and having junior faculty present opinions before senior faculty. However, as one of the goals of this article was to identify papers pertinent to junior faculty, the inclusion of junior faculty helped create a more well-rounded discussion during the modified Delphi process and a more thorough approach to paper selection. Although we included a faculty member from an outside institution, our panel was primarily from a single institution, which may limit generalizability to programs with different demographic makeup and institutional culture. 

## Conclusions

IP affects a significant proportion of physicians and is linked to severe consequences. As IP is associated with burnout and suicidal ideation, the implications both for physician health and public well-being are startling. Here, we present five key papers relevant to both junior and senior EM educators who would like to better understand IP. The selected articles provide an overview of the pervasiveness of IP, present some protective but underutilized interventions, and advocate for cultural change. The literature on effective interventions is promising but not well developed. We call for significant resources dedicated to studying IP among EPs, with an aim toward understanding the factors leading to IP, its causal association with negative effects, and validation of effective intervention strategies. 

## Appendices

Search strategy

(("impostorism"[ti] OR "imposterism"[ti] OR "impostor syndrome"[ti] OR "imposter syndrome"[ti] OR "impostor phenomenon"[ti] OR "imposter phenomenon"[ti]) AND ("physician"[tiab] OR "physicians"[tiab] OR "trainee"[tiab] OR "trainees"[tiab] OR "MD"[tiab] OR "MDs"[tiab] OR "learner"[tiab] OR "learners"[tiab] OR "doctor"[tiab] OR "doctors"[tiab] OR "intern"[tiab] OR "interns"[tiab] OR "student"[tiab] OR "students"[tiab]) AND ("medicine"[tiab] OR "medical"[tiab]))

## References

[1] Clance PR, Imes SA (1978). The imposter phenomenon in high achieving women: dynamics and therapeutic intervention. Psychol Psychother: Theory Res Pract.

[2] Batur A, Aksan A, Meneksedag Y, Karaca MA (2023). Impostor phenomenon and burnout syndrome among emergency physicians: a cross-sectional study. Arch Environ Occup Health.

[3] LaDonna KA, Ginsburg S, Watling C (2018). "Rising to the level of your incompetence": what physicians’ self-assessment of their performance reveals about the imposter syndrome in medicine. Acad Med.

[4] Bravata DM, Watts SA, Keefer AL (2020). Prevalence, predictors, and treatment of impostor syndrome: a systematic review. J Gen Intern Med.

[5] Gottlieb M, Chung A, Battaglioli N, Sebok-Syer SS, Kalantari A (2020). Impostor syndrome among physicians and physicians in training: a scoping review. Med Educ.

[6] Quinn A, Gottlieb M, Chan TM (2019). Curated collections for educators: five key papers on clinical teaching. Cureus.

[7] Chodoff A, Conyers L, Wright S, Levine R (2023). "I never should have been a doctor": a qualitative study of imposter phenomenon among internal medicine residents. BMC Med Educ.

[8] Shanafelt TD, Dyrbye LN, Sinsky C (2022). Imposter phenomenon in US physicians relative to the US working population. Mayo Clin Proc.

[9] Rehsi AS, McCarthy KE (2024). Twelve tips for recognizing and supporting medical learners experiencing impostorism. Med Teach.

[10] Siddiqui ZK, Church HR, Jayasuriya R, Boddice T, Tomlinson J (2024). Educational interventions for imposter phenomenon in healthcare: a scoping review. BMC Med Educ.

[11] Morgenstern BZ, Beck Dallaghan G (2021). Should medical educators help learners reframe imposterism?. Teach Learn Med.

[12] Rivera N, Feldman EA, Augustin DA, Caceres W, Gans HA, Blankenburg R (2021). Do I belong here? Confronting imposter syndrome at an individual, peer, and institutional level in health professionals. MedEdPORTAL.

[13] Thomas M, Bigatti S (2020). Perfectionism, impostor phenomenon, and mental health in medicine: a literature review. Int J Med Educ.

[14] Franchi T, Russell-Sewell N (2023). Medical students and the impostor phenomenon: a coexistence precipitated and perpetuated by the educational environment?. Med Sci Educ.

[15] Freeman J, Peisah C (2022). Imposter syndrome in doctors beyond training: a narrative review. Australas Psychiatry.

[16] Ogunyemi D, Lee T, Ma M, Osuma A, Eghbali M, Bouri N (2022). Improving wellness: defeating impostor syndrome in medical education using an interactive reflective workshop. PLoS One.

[17] Clance PR, OToole MA (1987). The imposter phenomenon: an internal barrier to empowerment and achievement. Women Ther.

[18] Gottlieb M (2023). When I say … imposter syndrome. Med Educ.

[19] Neufeld A, Babenko O, Lai H, Svrcek C, Malin G (2023). Why do we feel like intellectual frauds? A self-determination theory perspective on the impostor phenomenon in medical students. Teach Learn Med.

[20] Villwock JA, Sobin LB, Koester LA, Harris TM (2016). Impostor syndrome and burnout among American medical students: a pilot study. Int J Med Educ.

[21] Legassie J, Zibrowski EM, Goldszmidt MA (2008). Measuring resident well-being: impostorism and burnout syndrome in residency. J Gen Intern Med.

[22] Rosenthal S, Schlussel Y, Yaden MB, DeSantis J, Trayes K, Pohl C, Hojat M (2021). Persistent impostor phenomenon is associated with distress in medical students. Fam Med.

[23] Jefferson FA, Fadel A, Findlay BL (2024). The prevalence of impostor phenomenon and its association with burnout amongst urologists. BJU Int.

[24] Chakraverty D, Cavazos JE, Jeffe DB (2022). Exploring reasons for MD-PhD trainees' experiences of impostor phenomenon. BMC Med Educ.

[25] Joseph B, Tseng ES, Zielinski MD (2023). Feeling like an imposter: are surgeons holding themselves back?. Trauma Surg Acute Care Open.

[26] Leach PK, Nygaard RM, Chipman JG, Brunsvold ME, Marek AP (2019). Impostor phenomenon and burnout in general surgeons and general surgery residents. J Surg Educ.

[27] Bhama AR, Ritz EM, Anand RJ, Auyang ED, Lipman J, Greenberg JA, Kapadia MR (2021). Imposter syndrome in surgical trainees: Clance Imposter Phenomenon Scale assessment in general surgery residents. J Am Coll Surg.

[28] Stephens MB (2022). Taking off the mask: impostorism and medical education. PRiMER.

[29] Rudolph JW, Simon R, Rivard P, Dufresne RL, Raemer DB (2007). Debriefing with good judgment: combining rigorous feedback with genuine inquiry. Anesthesiol Clin.

[30] Chen C (2020). Doctor who? Reflecting on impostor syndrome in medical learners. Can Fam Physician.

